# Prenatal antibiotics exposure does not influence experimental allergic asthma in mice

**DOI:** 10.3389/fimmu.2022.937577

**Published:** 2022-08-10

**Authors:** Imke Lingel, Adrienne N. Wilburn, Julie Hargis, Jaclyn W. McAlees, Yves Laumonnier, Claire A. Chougnet, Hitesh Deshmukh, Peter König, Ian P. Lewkowich, Inken Schmudde

**Affiliations:** ^1^ Institute of Anatomy, University of Lübeck, Lübeck, Germany; ^2^ Airway Research Center North (ARCN), Member of the German Center for Lung Research (DZL), Lübeck, Germany; ^3^ Division of Immunobiology, Cincinnati Children’s Hospital Medical Center, Cincinnati, OH, United States; ^4^ Immunology Graduate Program, University of Cincinnati, Cincinnati, OH, United States; ^5^ Institute for Systemic Inflammation Research, University of Lübeck, Lübeck, Germany; ^6^ Department of Pediatrics, University of Cincinnati, Cincinnati, OH, United States; ^7^ Division of Neonatology and Pulmonary Biology, Cincinnati Children’s Hospital Medical Center, Cincinnati, OH, United States

**Keywords:** asthma, microbiome, dysbiosis, ILCs, Th17 response, RORγt

## Abstract

Changes in microbiome (dysbiosis) contribute to severity of allergic asthma. Preexisting epidemiological studies in humans correlate perinatal dysbiosis with increased long-term asthma severity. However, these studies cannot discriminate between prenatal and postnatal effects of dysbiosis and suffer from a high variability of dysbiotic causes ranging from antibiotic treatment, delivery by caesarian section to early-life breastfeeding practices. Given that maternal antibiotic exposure in mice increases the risk of newborn bacterial pneumonia in offspring, we hypothesized that prenatal maternal antibiotic-induced dysbiosis induces long-term immunological effects in the offspring that also increase long-term asthma severity. Therefore, dams were exposed to antibiotics (gentamycin, ampicillin, vancomycin) from embryonic day 15 until birth. Six weeks later, asthma was induced in the offspring by repeated applications of house dust mite extract. Airway function, cytokine production, pulmonary cell composition and distribution were assessed. Our study revealed that prenatally induced dysbiosis in mice led to an increase in pulmonary Th17^+^ non-conventional T cells with limited functional effect on airway resistance, pro-asthmatic Th2/Th17 cytokine production, pulmonary localization and cell-cell contacts. These data indicate that dysbiosis-related immune-modulation with long-term effects on asthma development occurs to a lesser extent prenatally and will allow to focus future studies on more decisive postnatal timeframes.

## Introduction

Several risk factors ranging from genetic predisposition, increasing allergen prevalence, decline in parasite infections and changes in microbial colonization are known to contribute to the increasing asthma prevalence particularly in developed countries. These factors critically drive asthma development or asthma severity at different developmental stages. Although maternal exposures have been implicated in long term development of inflammatory diseases, this might be through altering the trajectory over which microbial colonization in offspring occurs. This trajectory is influenced by the transfer of microbiota that starts *in utero via* pioneer microbiota that settle in the meconium and the fetal gut (1–6). These microbiota resemble either vaginal or oral microbiome suggesting that they cross the placental barrier and subsequently colonize the fetus by translocation from vagina or following oral dissemination (2, 5, 6). During and after birth these pioneer skin and gut microbiota are replenished and displaced by bacteria transmitted during passage through the birth canal and/or breast feeding (7–12). Within the first two years of life the microbiome undergoes further choreographed consolidation driven by environmental factors and nutrition, finally reaching the mature colonization pattern (13–16). Thus, early life exposures during this “neonatal window of opportunity” can change the trajectory through which this mature colonization pattern is established. This results in variable interactions between microbiota and host, and is thought to play a critical role in establishing life-long homeostasis (17, 18). However, whether changes to the maternal microbiome during pregnancy are sufficient to alter asthma risk in offspring *via* gut-lung axis remains unclear.

Analyzing the modulation of asthma risk by changes of the microbiome requires basal understanding of immune cells involved in asthma development. Severe allergic asthma is synergistically driven by maladaptive T helper (Th) 2 and Th17 immune responses against harmless air-born allergens presented by dendritic cells. Together these pathways initiate a mixed eosinophilic/neutrophilic inflammation driven by IL-5 and IL-17, respectively (19). Also airway hyperreactivity (AHR), airway remodeling and mucus production are modulated by combined Th2/Th17 immune responses (20–22). Other cellular components are lymphoid-derived cells like innate lymphoid cells (ILC) and non-conventional T cells, which contribute to immediate inflammatory responses expression of cytokines and receptors, phagocytosis capacitites and release of toxic granules (23, 24). ILCs originate from a common lymphoid progenitor, express similar cytokines as T cells, but do not express the T cell receptor. They reside in the tissues and contribute to inflammation by cytokine release (25, 26). Among the ILC subpopulations, ILC2 and ILC3 contribute to an allergic asthmatic phenotype by secretion of IL-5/IL-13 and IL-17A, respectively (27–29). Non-conventional T cells comprise γδ T cells, invariant NKT cells and mucosal-associated invariant T cell (30). In contrast to conventional T cells they do not express an αβ T cell receptor and rather contribute to innate immunity, since they lack the ability to induce allergen-specific B cell activation (31). They can rapidly respond to pathogens *via* IL-17A secretion In the context of allergic asthma they have the potential to fuel Th2/Th17 induced asthma responses (32, 33). To date it is not clear whether any of these cells are long-term regulated in allergic asthma by early-life changes in microbiome.

In order to test whether changes to the maternal microbiome during late pregnancy are sufficient to alter asthma risk in offspring by modulating lung immune responses, pregnant mice were given antibiotics (Abx) during late pregnancy, to induce dysbiosis in offspring. Control and dysbiosis-exposed offspring were weaned and subsequently exposed to the allergen house dust mite (HDM) in a well-described mouse model of allergic asthma. Compared to control HDM-exposed animals, dysbiosis-exposed offspring subsequently exposed to HDM displayed an increase in the frequency of pulmonary IL-17A^+^ non-conventional T cells localized around airways. However, this altered IL-17A^+^ non-conventional T cell frequency was not associated with any functional effect on airway resistance and pro-asthmatic cytokine production. Establishing a staining protocol for immunohistochemistry (IHC) enabled us further demonstrate that prenatal antibiotic exposure does not alter Th17 cell localization as indicated by RORγt staining – either in the airways or in relation to pulmonary CD11c^+^ antigen presenting cells. Our study suggests that prenatal maternal Abx treatment has insignificant effects on the asthma phenotype in the offspring, indicating that the postnatal colonization may be more important for the pathogenesis of allergic asthma. Thereby, our study will allow to focus future studies on more decisive postnatal timeframes.

## Methods

### Mice

C57BL/6 mice (in-house breeding) used for establishing microscopic analyses were maintained in the University of Lübeck specific pathogen-free facility and used at 8-12 weeks of age. Animal care was provided in accordance with German animal protection laws. Studies using these mice were reviewed and approved by the Schleswig-Holstein state authorities (registration number 39(44-5/18)). A/J, A/J RORC-GFP as well as C57BL/6 mice (in-house breeding) used for *in vivo* HDM immunization were maintained in the AAALAC-accredited CCHMC animal facility and used at 6-8 weeks of age. Studies using these mice were reviewed and approved by the CCHMC Institutional Animal Care and Use Committee (registration number 2013-0180). All mice were kept under specific-pathogen-free conditions and received sterile food and drinking water *ad libitum*.

### Animal handling

Experiments including Abx treatment and asthma induction were performed in the CCHMC animal facility. For breeding, one male and 2-3 female C57BL/6 mice were mated overnight, and the following morning the presence of vaginal plugs was assessed. For confirmed pregnant female mice, on embryonic day 15 (E15) drinking water was replaced with water containing 0.5 % sucralose, or 0.5 % sucralose with ampicillin, gentamycin and vancomycin (all from Gold Biotechnology Inc.; all at 1.0 mg/mL) as previously published (34). After birth, Abx treatment was discontinued immediately. Offspring of both biological sexes were weaned at the age of 3-4 weeks. Litter sizes ranged from 3 to 10 pups. At 6-8 weeks, asthma was induced in control and Abx-exposed offspring. Mice were immunized intraperitoneally (i.p.) with 10 µg/100 µL of HDM (Greer Laboratories) on experimental day 0 and 7 dissolved in phosphate-buffered saline (PBS; pH7.4; 685 mM NaCl, 13.5 mM KCl, 50 mM NaH_2_PO_4_*H_2_O, 10 mM Na_2_HPO_4_*H_2_O; all from Carl Roth GmbH). Mice originating from one litter were split into both asthma and control group. After 14 and 21 days, mice were treated with 100 µg/40 µL HDM intratracheally (i.t.). Intratracheal treatments were carried out under general anesthesia with ketamine (provided by the veterinary service of CCHMC; 120 mg/kg bodyweight) and xylazine (provided by the veterinary service of CCHMC; 8 mg/kg bodyweight). Control mice received a comparable treatment using PBS. 72 h after the last treatment, lung function was assessed using the flexiVent system (SciReq) and the asthma phenotype was assessed.

### Measurement of allergen-induced AHR

AHR was measured in mice anesthetized with sodium pentobarbital (provided by the veterinary service of CCHMC; 70 mg/kg body weight) and xylazine (10 mg/kg body weight). Briefly, the mouse trachea was exposed by surgical incision of the skin, submaxillary glands and muscles surrounding the trachea. Trachea was cannulated using a 20 G cannula that was inserted and tightened by surgical suture. Mice were mechanically ventilated using a flexiVent System at a positive end-expiratory pressure of 3 cm H_2_O with 150 breaths per minute. Complete muscle relaxation was induced by i.p. injection of pancuronium bromide (Sigma-Aldrich Inc.; 1 mg/kg body weight). Aerosolized Acetyl-β-Methyl-Choline (methacholine; Sigma-Aldrich Inc.) was generated by an ultrasonic nebulizer and delivered with increasing doses (0, 12.5, 50, 100 mg/ml) in-line through the inhalation port for 10 s. Airway resistance (R_RS_) was measured 2 minutes later.

### Collection of bronchoalveolar lavage (BAL) fluid

After lung function measurement, lungs were lavaged three times with 1 mL ice-cold Hank’s Balanced Salt Solution (HBSS; Thermo Fisher Scientific Inc.). To isolate BAL cells, samples were centrifuged (300g, 6 min) and erythrocytes lysed using ACK buffer (Lonza AG). Lysis was stopped by adding ice-cold PBS containing 5 % fetal bovine serum (FBS; Thermo Fisher Scientific) and subsequent centrifugation (300g, 6 min). Total BAL cell counts were determined using a Neubauer chamber. 5-10 x 10^4^ cells were transferred to objective slides by cytospin centrifugation (400 rpm, 4 min). Cytospins were stained with DiffQuick stain (RAL Diagnostics) and 500 cells were morphologically differentiated by light microscopy.

### Isolation of pulmonary cells and cytokine measurements

The postcaval lobe was removed and snap frozen for mRNA isolation. Other lobes were minced and incubated in 7 mL RPMI 1640 (Gibco Life Technologies Corp.) containing Liberase™ Research Grade (Sigma-Aldrich Inc.; 1.5 mg/mL), DNAse I (Roche AG; 0.5 mg/mL) and Penicillin/Streptomycin/L-Glutamine (Gibco Life Technologies Corp.; 1 % v/v) for 45 min at 37°C. Single cell suspensions were prepared by passing minced lobes through a 70 µm strainer. Lung cells were centrifuged (400g, 8 min) and erythrocytes were lysed by incubation with ACK buffer (3 min). Cell numbers were determined using a Neubauer chamber and adjusted to 4 x 10^6^ cells/mL and used for cytokine measurements and flow cytometry.

### Cytokine measurement

1 x 10^6^ lung cells were restimulated *ex vivo* with 0.03 mg/mL HDM in RPMI 1640 (10 % FBS, 1 % Penicillin/Streptomycin, 0.1 % β-Mercaptoethanol (Thermo Fisher Scientific Corp.)) for 72 h at 37°C. Supernatants were harvested. Production of interleukin (IL)-4, IL-5, IL-13 and IL-17A in culture supernatants was determined using matched antibody pairs from Invitrogen. ELISA plates were coated with 50 µL of diluted antibodies (IL-4: clone 11B11; IL-5: clone TRFK5; IL-13: clone eBio13A; IL-17A: clone eBio17CK15A5) overnight at 4°C. 1:2 Standard dilution series with a minimum concentration of 4.88 pg/mL were prepared using recombinant IL-4, IL-5, IL-13 and IL-17A (all Invitrogen). Plates were washed and 50 µL of sample were applied overnight at 4°C. Subsequently, plates were washed and biotinylated antibodies (IL-4: clone BVD6-24G2; IL-5: clone TRFK4; IL-13: clone eBio16H; IL-17A: clone eBio17B7) were added for 2 h at RT. Plates were washed and HRP-Avidin (Invitrogen) was added for 30 min at RT. After a final washing step Super AquaBlue Invitrogen™ (Invitrogen Inc.) was added to the wells, and after developing time of 8 - 30 min (until concentration gradient had fully developed) optical density of ELISA plates was determined with a BioTek plate reader (BioTek Instruments Inc.).

### Intracellular cytokine measurement by flow cytometry

2.5 x 10^5^ lung cells were restimulated *ex vivo* in 250 µL Iscove’s modified Dulbeccos’s Medium (IMDM; Thermo Fisher Scientific Inc.) with Phorbol 12-myristate 13-acetate (PMA)/ionomycin (all Sigma-Aldrich Inc.; 50 ng/mL and 500 ng/mL) and 1x Brefeldin/Monensin (Sigma-Aldrich Inc.) for 4 h at 37°C. Cells were centrifuged (400g, 3 min) and the pellet was resuspended in PBS/bovine serum albumin (BSA; 0.5 %; Sigma-Aldrich Inc.). Nonspecific antibody binding was blocked using anti-CD16/CD32 Mouse BD Fc-block™ (Becton, Dickinson Inc.) for 15 min at 37°C. Cells were stained with anti-CD90.2-BV605 (clone 53-2.1, BioLegend Inc.), anti-TCRβ-AF700/AF488 (clone H57-597, BioLegend Inc.) and anti-CD3ϵ-APC-Cy7 (clone eBio500A2, Invitrogen Inc.) for 30 min at 37°C. Cells were washed and BD Horizon Viability Dye V500 (Becton Dickinson Inc.) was added in PBS in darkness for 15 min at RT. Cells were washed and then fixed/permeabilized using Cytofix/Cytoperm™ (Becton Dickinson Inc) in darkness for 60 min at RT. After additional blocking with Fc-Block, intracellular staining with anti-IL-17A-PE (clone eBio17B7; Invitrogen Inc.) for 30 min at 4°C was performed. Flow cytometric measurements were performed using a BD™ LSR II flow cytometer (Becton Dickinson Inc.). IL-17A^+^ cells were analyzed as depicted in **Supplementary Figure 1**.

### RNA extraction

RNA from the postcaval lobe was isolated using TRIzol™ reagent (Thermo Fisher Scientic Inc.) according to the manufacturer’s instructions. Reverse transcription was performed using random primers (Invitrogen Inc.), dNTP mix (Thermo Fisher Scientific Inc.), 1^st^ strand buffer (Thermo Fisher Scientific Inc.), Dithiothreitol (USP grade; Thermo Fisher Inc.), RNase out (New England BioLabs Inc.), Superscript™ II Reverse Transcriptase (Invitrogen Inc.). Quantitative PCR was done using Light Cycler 480^®^ SYBR Green I Master Mix (Roche AG) on a LightCycler^®^ 480 Instrument II (Roche) using the following primers (Bio-Rad Laboratories Inc.): *S14* 5’-GAG GAG TCT GGA GAC GAC GA-3’ (sense) and 5’-TGG CAG ACA CCA CCA ACA TT-3’ (antisense), *Il17a* 5’-CAG ACT ACC TCA ACC GTT CCA C-3’ (sense) and 5’-TCC AGC TTT CCC TCC GCA TTG A-3’ (antisense), *Il17f* 5’-TGC CAG GAG GTA GTA TGA AGC TT-3’ (sense) and 5’-ATG CAG CCC AAG TTC CTA CAC T-3’ (antisense), *Il22* 5’-ACG CAA GCA TTT CTC AGA GA-3’ (sense) and 5’-AAC ATG AGT CCA GGG AGA GC-3’ (antisense), *Mpo* 5’-TCC CAC TCA GCA AGG TCT T-3’ (sense) and 5’-TAA GAG CAG GCA AAT CCA G-3’ (antisense), *Muc5b* 5’-TGT ACT GCC CCC AGG ATG GGC-3’ (sense) and 5’-AGC TCA GCT CTG CCT GAC CCT-3’ (antisense), *Muc5ac* 5’-CCA TGC AGA GTC CTC AGA ACA A-3’ (sense) and 5’-TTA CTG GAA AGG CCC AAG CA-3’ (antisense), *Gob5* 5’-ACT AAG GTG GCC TAC CTC CAA-3’ (sense) and 5’-GGA GGT GAC AGT CAA GGT GAG A-3’ (antisense).

### Tissue removal and cryosectioning

The small intestines of A/J WT and RORC-GFP mice were removed and 10 cm of intestine were rinsed with PBS. The intestinal lumen was filled with 1 % Paraformaldehyde (PFA; Agar Scientific Ltd.), coiled into a spiral and placed between two sponges in a tissue cassette. Tissue was fixed using Cytofix/Cytoperm™ (Becton Dickinson Inc.) 1:4 diluted with PBS overnight at 4°C. Tissue was washed with PBS for 8 h at 4°C. Embedding was prepared by incubation in 30 % sucrose solution (Carl Roth GmbH) diluted 1:1 with TissueTek^®^ O.C.T Compound™ (Science Services GmbH) overnight at 4°C. Tissue was embedded in TissueTek^®^ O.C.T Compound™ and frozen in liquid nitrogen. 18 µm cryosections were generated and used for IHC staining.

To examine pulmonary histology, lungs were perfused *via* the right ventricle with 3 mL ice cold Ringer solution (ph7.4; 5.6 mM KCl, 136.4 mM NaCl, 1 mM MgCl_2_*6 H_2_O, 2.2 mM CaCl_2_*2 H_2_O; all Carl Roth GmbH; 11 mM Glucose, 10 mM HEPES; both Sigma-Aldrich Inc.) containing 5 % heparin (Braun). 3 % LM agarose (Universal Medical Inc.) in Ringer solution at 37°C was injected into the lungs *via* the trachea. Filled lungs were removed and transferred to ice cold Ringer solution. 300 µm sections were sliced using a vibratome, fixed with 1 % PFA in PBS for 15 min at RT while shaking, washed three times in PBS each for 15 min at RT while shaking, incubated with 20 % sucrose overnight at 4°C and used for IHC staining.

### Immunofluorescence staining

Lung or intestinal sections were incubated with IHC blocking buffer (PBS, 0,3 % v/v Triton X-100 (Thermo Fisher Scientific Inc.), 10 % v/v normal mouse serum (Invitrogen Inc.)) for 20 min at RT. Sections were incubated in IHC blocking buffer with purified anti-RORγt (AFKJS-9; Invitrogen Inc.) and where indicated with anti-α-SMA-FITC (1A4; Invitrogen Inc.), anti-TCRβ-AF647/AF488 (H57-597; BioLegend Inc.) or anti-CD11c-AF647 (N418; BioLegend Inc.) diluted in PBS (overnight (intestinal sections) or 60 h (lung sections), 4°C). After three washing steps, sections were incubated with secondary polyclonal anti-rat IgG-DyLight-549/AF647 (Jackson ImmunoResearch Inc.) in PBS supplemented with 5 % normal mouse serum (Invitrogen Inc.) for 60 min at RT. For double-labeling with CD3, an additional blocking step with 10 % normal rat serum (Linaris Biologische Produkte) was performed for 1h at RT, followed by incubation with anti-CD3-AF647 (17A2; BioLegend Inc.) for 2h, at RT. Slices were rinsed three times and cover slipped with Mowiol mounting medium (12 g Mowiol 4-88 (Sigma-Aldrich), 30 mL A. bidest., 60 mL Tris buffer (0.2 M; Invitrogen Inc.), 30 g Glycerin (pH 8.5; Merck)) with or without 1 µg/mL Hoechst dye (Thermo Fisher Scientific Inc.).

### Laser scanning confocal microscopy

Stained slices were evaluated with confocal microscopy. Z-stacks (40 μm with a resolution of 1 μm) were recorded to allow a three-dimensional presentation of lung structures including possible cell interactions. Airway and blood vessels were identified by the use of anti-α-SMA-FITC (1A4; Invitrogen) or using autofluorescence (excitation: 488nm; detection: 490-530 nm). Analysis was performed using a laser scanning confocal microscope Zeiss 710 meta (Carl Zeiss AG).

### Statistical analysis

Statistical analysis was performed using the GraphPad Prism version 5 (GraphPad Software Inc.). Outliers were excluded after Grubbs test. Data are represented as mean and standard error of the mean (SEM). Statistical differences for the comparison of multiple independent treatment groups were analyzed for normality using D’Agostino and Pearson omnibus normality Test. Gaussian distributed samples were evaluated by one-way ANOVA followed by Tukey Post Test. Non-Gaussian samples were evaluated by Kruskal-Wallis-Test and Dunns’s Post Test. For comparison of multiple dependent measurements two-way ANOVA followed by Bonferroni Post Test was chosen. Statistical difference between two groups was evaluated by Student T Test after passing D’Agostino and Pearson omnibus normality Test or Mann-Whitney-U-Test. p values < 0.05 were considered statistically significant.

## Results

### Prenatally induced dysbiosis of dams increases recruitment of IL-17A producing cells to the lung in allergen-exposed offspring

**Figure 1:**
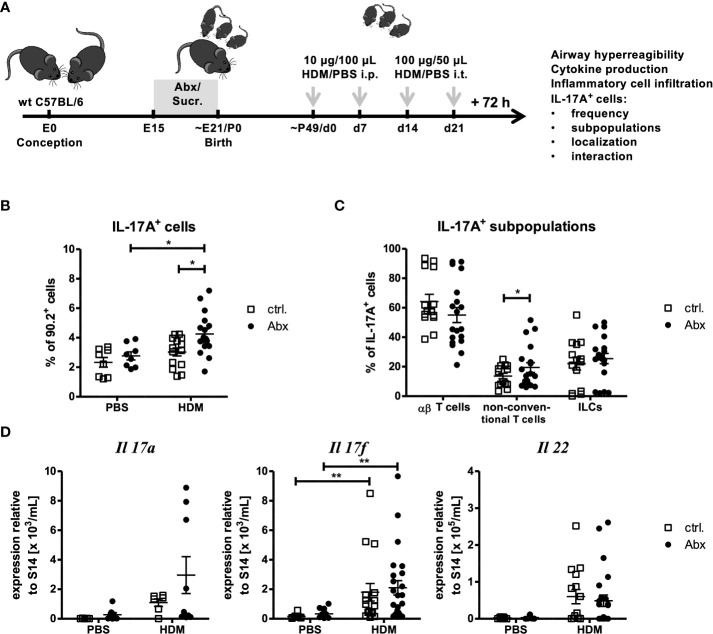
Prenatal antibiotic exposure increases the frequency of IL-17A^+^ pulmonary cells without affecting gene expression. **(A)** C57BL/6 mice were treated with vancomycin, ampicillin and gentamycin (all at 1.0 mg/mL)(Abx) from prenatal day 15 until birth. Mice treated with sucralose served as controls (ctrl.). At 6-8 weeks, offspring C57BL/6 mice were immunized i.p. with 10 µg/100 µL of HDM. Seven days later mice received an additional i.p. injection of HDM. After 14 and 21 days mice were treated with 100 µg/40 µL HDM i.t. Mice treated with PBS served as non-asthmatic controls (B, D). 72 h after the last treatment the asthma phenotype was assessed. **(B)** Single lung cell suspensions were restimulated with PMA (50 ng/mL) and ionomycin (0.5 mg/mL). After intracellular staining of IL-17A, CD90.2^+^ lymphocytes were analyzed regarding IL-17A production by flow cytometry. **(C)** To further differentiate IL-17A^+^ populations, CD3 and TCRβ expression was analyzed to identify CD3^+^TCRβ^+^ αβ T cells, CD3^+^TCRβ^-^ non-conventional T cells and CD3^-^TCRβ^-^ ILCs. **(D)** Further, 72 h after the last *in vivo* treatment RNA expression of Th17-related cytokines normalized to the expression of the housekeeping gene S14 by pulmonary cells was assessed. Scatter plot with mean ± SEM; n = 5-25 mice. Each dot represents one mouse. Differences between groups were tested by one-way ANOVA (after passing D’Agostino and Pearson omnibus normality Test) and Tukey Post Test)(B), Student T Test (after passing D’Agostino and Pearson omnibus normality Test)(C)or Kruskal-Wallis-Test and Dunns’s Post Test for significance (D); * p < 0.05, ** p < 0.01.

To date, human and mouse studies tried to correlate early life dysbiosis with long-term asthma susceptibility and severity. However, it remains unclear when and how dysbiosis most strongly influences asthma outcomes. To determine if prenatal maternal dysbiosis is sufficient to induce changes in allergen-induced cellular recruitment, we treated dams from E15 until birth with a cocktail of three Abx (Ampicillin, Vancomycin and Gentamicin) in sucralose-sweetened drinking water, or sucralose-sweetened drinking water alone (**Figure 1A**). After birth, mothers were switched to conventional water. This model has been shown to induce profound neonatal dysbiosis and increased susceptibility to neonatal pneumonia (34). At 6-8 weeks of age, animals were exposed to an extract of the aeroallergen HDM. To this end, animals were administered two doses of 100 µg of HDM in saline, or saline alone i.p. one week apart. Starting one week after the final i.p HDM exposure, animals were given two doses of i.t. 100 µg HDM one week apart. 72 hours after the final HDM exposure, recruitment of cytokine-producing cells to the airways was measured. To quantify *in situ* cytokine production in the lung at the time of sacrifice, we tested the capacity of lung cells to accumulate IL-13 and IL-17A in response to PMA/ionomycin in presence of Brefeldin/monensin by flow cytometry. After dead cell exclusion lymphocytes were identified as CD90.2^+^ cells (**Supplementary Figure 1**). As expected, the compartment of IL-13- and IL-17A-expressing cells was slightly increased in lung cells purified from HDM immunized lungs. Although the number of IL-13-expressing cells was not further regulated by prenatal antibiotic exposure (**Supplementary Figure 2**), the frequency and number of IL-17A-expressing lung cells were significantly increased in the HDM-treated offspring of Abx-treated dams (**Figure 1B**, **Supplementary Figure 3A**). Further analyses of the IL-17A^+^ cell compartment following a gating scheme considering TCRβ and CD3 expression (**Supplementary Figure 1**) allowed the identification of ILCs (CD3^-^TCRβ^-^), αβ T cells (CD3^+^TCRβ^+^) and non-conventional T cells (CD3^+^TCRβ^-^). IL-17A^+^ cells were predominantly (>50 %) αβ T cells (**Figure 1C**). ILCs and non-conventional T cells accounted each for approximately 20 % of IL-17A^+^ lung cells. Prenatal treatment with Abx did not affect the frequency of IL-17A^+^CD4^+^ T cells, or ILCs following HDM-exposure, whereas the frequency of non-conventional T cells was significantly increased in offspring of HDM-exposed, Abx-treated dams. In terms of absolute cell numbers all three subpopulations were significantly increased due to the increase of total IL-17A^+^ cells (**Supplementary Figure 3B**).

Given the increase in IL-17A^+^ cells, the expression of Th17-related cytokines *Il17a*, *I17f* and *Il22* was analyzed in whole lung homogenates (**Figure 1D**). A trend towards increased gene expression of *Il17a*, *I17f* and *Il22* was observed upon HDM immunization compared to PBS-treated control mice. Further, although prenatal Abx treatment tended to increase the expression of *Il17a* and *Il17f* in asthmatic mice, expression of *IL22* was not regulated by prenatal Abx treatment. Since IL-17 is known to regulate the migration of neutrophils and activate airway mucus production we analyzed the expression of neutrophil-associated genes (*Mpo*) and mucus-associated genes (*Muc5b*, *Muc5ac* and *Gob5)*. No significant Abx-mediated regulation of these genes was observed in HDM-exposed mice (**Supplementary Figures 4A, B**). In summary prenatal Abx treatment increased the number of non-conventional T cells capable of producing IL-17A^+^ cells in the lung, but did not directly induce pulmonary neutrophilia, or augment mucus production.

### Prenatally induced maternal dysbiosis does not alter allergen-induced asthma development in offspring

**Figure 2:**
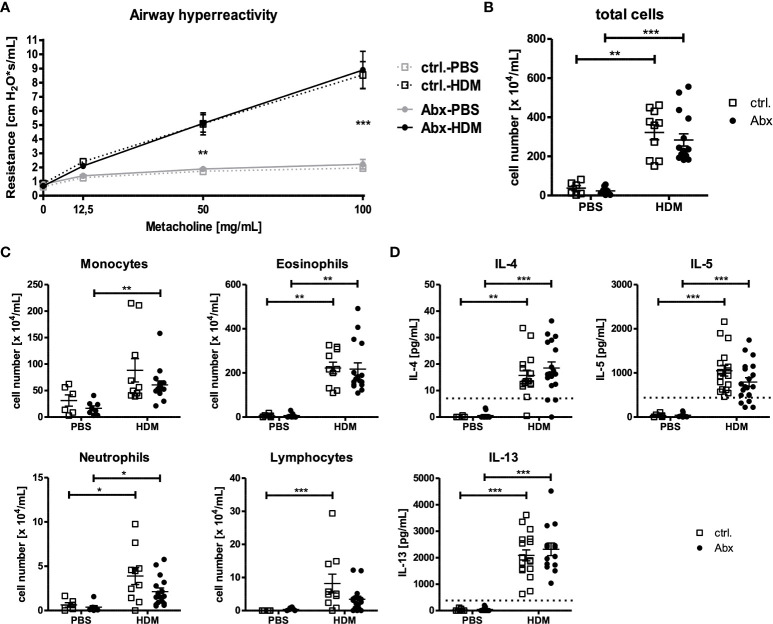
Prenatal antibiotic exposure of dams does not influence the asthma phenotype of the offspring. C57BL/6 mice were treated with vancomycin, ampicillin and gentamycin (all at 1.0 mg/mL)(Abx) from prenatal day 15 until birth. Mice treated with sucralose served as controls (ctrl.). At 6-8 weeks, offspring C57BL/6 mice were immunized i.p. with 10 µg/100 µL of HDM. Seven days later mice received an additional i.p. injection of HDM. After 14 and 21 days, mice were treated with 100 µg/40 µL HDM i.t. Mice treated with PBS served as non-asthmatic controls. 72 h after the last treatment AHR in response to i.t. administration of methacholine measured as airway resistance using Flexivent **(A)**, cellular infiltration of the airways **(B, C)** and cytokine production of single lung cell suspensions after 72 h *ex vivo* cell culture **(D)** were assessed. Scatter plot with mean ± SEM, n = 6-18 mice. Each dot represents one mouse. Dotted lines (D) indicate the detection limit. Differences between groups were tested by two-way ANOVA and Bonferroni Post Test (A) or Kruskal-Wallis-Test and Dunns’s Post Test (B–D) for significance; * p < 0.05, ** p < 0.01, *** p < 0.001.

Given observed changes in allergen-induced recruitment of IL-17A^+^ cells in dysbiosis exposed offspring, and the described role of IL-17A-producing cells in driving the development of asthma, we next examined the impact of prenatally induced dysbiosis on the asthma phenotype. As expected, HDM exposure induced marked changes in airway function in offspring of sucralose-exposed dams (**Figure 2A**). Offspring of dams treated with prenatal Abx developed comparable airway responses to HDM-treated offspring of control animals. Accordingly, the total number of inflammatory cells present in the BAL fluid (**Figure 2B**) was comparable in offspring of HDM-treated control dams, and offspring of Abx-treated dams. Similarly, the composition of the inflammatory infiltrate (monocytes, lymphocytes, eosinophils, neutrophils) was not different in HDM-exposed offspring of control dams, or offspring of Abx-exposed dams (**Figure 2C**). These data are complemented by the observation that HDM sensitization significantly increased the concentrations of the Th2 cytokines IL-4, -5 and -13 by pulmonary cells restimulated *ex vivo* with HDM independent of prenatal antibiotic exposure (**Figure 2D**). IL-17A was not measurably induced under any condition (data not shown). Taken together, although HDM immunization induced an asthmatic phenotype in the offspring, maternal dysbiosis did not alter these effects.

### Pulmonary cells expressing the IL-17A transcription factor RORγt can be identified *in situ*

**Figure 3:**
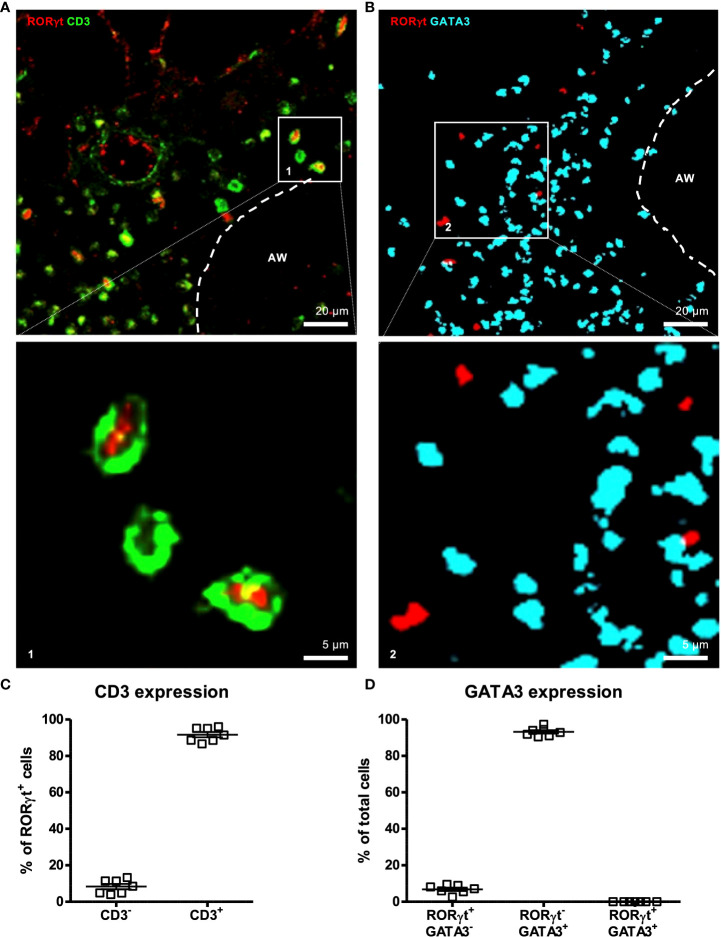
RORγt is suitable as a marker for pulmonary IL-17A^+^ cells *in situ*. At 6-8 weeks C57BL/6 mice were immunized i.p. with 10 µg/100 µL of HDM. Seven days later mice received an additional i.p. injection of HDM. After 14 and 21 days mice were treated with 100 µg/40 µL HDM i.t. 72 h after the last treatment 300 µm lung sections of agarose filled lungs were prepared and stained with anti-RORγt (red) and anti-CD3 (green) **(A)** or anti-RORγt (red) and anti-GATA3 (turquoise) **(B)**. **(C)** RORγt^+^ cells were analyzed regarding CD3 expression. **(D)** Total cells were analyzed regarding RORγt and GATA3 expression. Stained slices were evaluated with confocal microscopy. Abbreviations: AW, airway. Data are representative of two independent experiments. Scatter plot with mean ± SEM; n = 7 mice. Each dot represents the Z-stack of one mouse.

Based on the observation of Gray et al. (34) that dysbiosis interrupts migration of ILC3s to the lung by modulating the expression of CCR4, we aimed to investigate *via* IHC whether dysbiosis also influences the localization of IL-17A^+^ cells *in situ* or affects interactions with other immune cells. Since detection of cytokine signals is difficult by IHC (Brefeldin A-mediated inhibition of secretion is not possible in IHC), the key transcription factor of IL-17A^+^ cells RORγt was chosen as a marker for IL-17A expression. Using intestinal slides from A/J RORC-GFP reporter mice we tested the RORγt antibody for specificity and applicability in IHC (**Supplementary Figure 5**). Co-staining with anti-RORγt antibodies and Hoechst stain expectedly revealed a nuclear localization of the anti-RORγt (data not shown). In order to further prove specificity of the anti-RORγt antibody and to test suitability for lung staining, co-expression with CD3 in the lung was analyzed (**Figures 3A, C**). About 90 % of RORγt^+^ cells co-expressed the T cell marker CD3. Further, as a proof of concept less than 10 % of RORγt cells expressed the key Th2 transcription factor GATA3 (**Figure 3B, D**). In summary, staining with anti-RORγt allows identification of RORγt^+^ cells in the lung *in situ* without substantial overlap with the Th2 cell compartment.

### Prenatally induced dysbiosis of dams does not influence localization of offspring pulmonary RORγt^+^ cells

**Figure 4:**
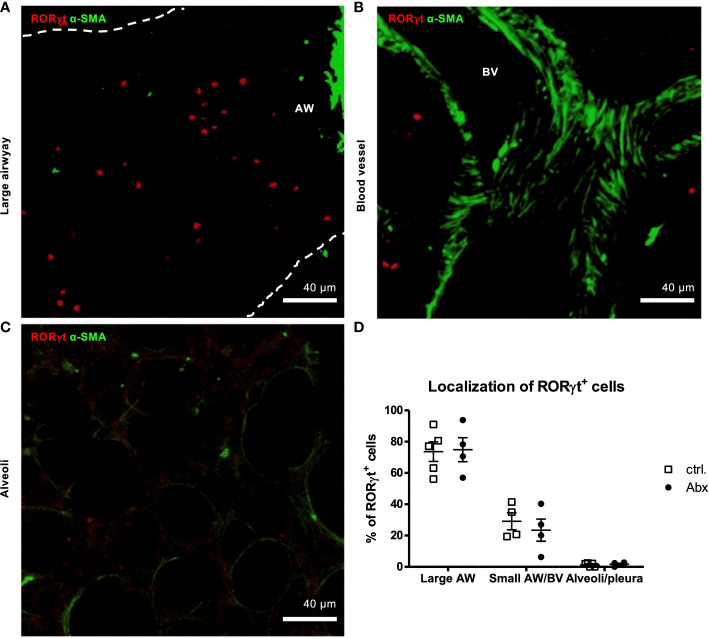
Prenatal antibiotic exposure of dams does not influence localization of offspring pulmonary RORγt^+^ cells *in situ*. C57BL/6 mice were treated with vancomycin, ampicillin and gentamycin (all at 1.0 mg/mL)(Abx) from prenatal day 15 until birth. Mice treated with sucralose served as controls (ctrl.). At 6-8 weeks, offspring C57BL/6 mice were immunized i.p. with 10 µg/100 µL of HDM. Seven days later mice received an additional i.p. injection of HDM. After 14 and 21 days mice were treated with 100 µg/40 µL HDM i.t. 72 h after the last treatment 300 µm lung sections of agarose filled lungs were prepared. **(A, B)** For visualization of airways and blood vessels anti-aSMA (green) was added. **(C)** Alveoli were identified by autofluorescence. **(D)** Stained slices were evaluated with laser scanning confocal microscopy generating Z-Stacks for localization of RORγt^+^ cells (red). Abbreviations: AW, airway; BV, blood vessel. Data are representative of at least four independent experiments. Scatter plot with mean ± SEM. n = 4-5 mice. Each dot represents the Z-stack of one mouse.

To determine whether prenatal Abx-induced dysbiosis affects later life distribution of pulmonary IL-17A-producing cells, we used the previously established method of α-SMA staining (35) and autofluorescence in combination with the stain of RORγt. This allowed discrimination between large airways, small airways/blood vessels and the pleura/alveolar region as confirmed by TCR-β staining (**Supplementary Figure 6**). A major proportion (>70 %) of pulmonary RORγt^+^ cells were localized around larger airways in HDM-exposed animals (**Figures 4A, D**). In contrast, ~29 % and <1 % were found around small airways/blood vessels and at the pleura/alveolar region, respectively (**Figures 4B** **–D**). Importantly, prenatally induced dysbiosis did not affect the pulmonary distribution of RORγt^+^ cells after HDM exposure (**Figure 4D**).

**Figure 5:**
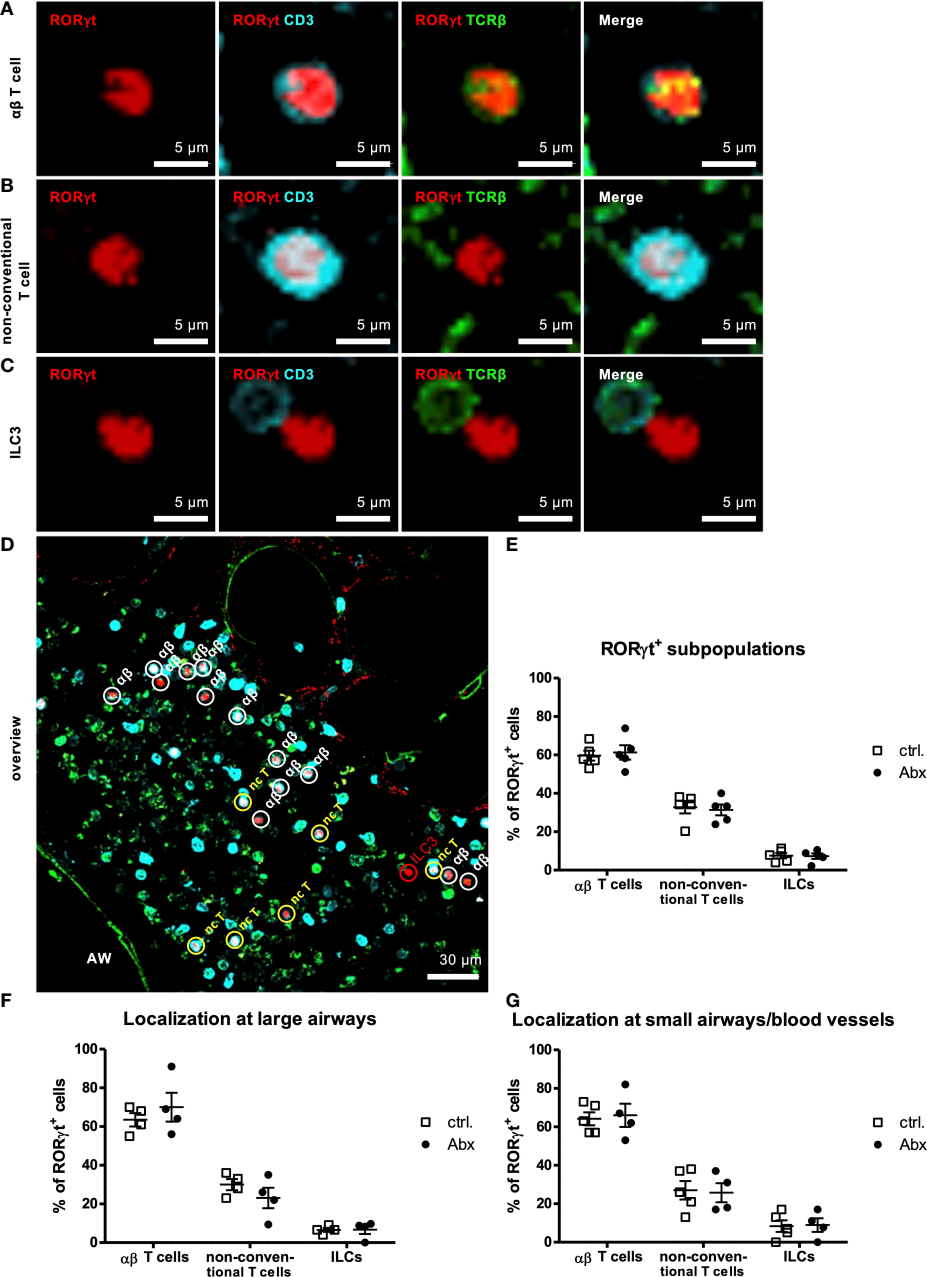
Prenatal antibiotic exposure of dams does not influence localization of offspring pulmonary RORγt^+^ subpopulations *in situ*. C57BL/6 mice were treated with vancomycin, ampicillin and gentamycin (all at 1.0 mg/mL)(Abx) from prenatal day 15 until birth. Mice treated with sucralose served as controls (ctrl.). At 6-8 weeks, offspring C57BL/6 mice were immunized i.p. with 10 µg/100 µL of HDM. Seven days later mice received an additional i.p. injection of HDM. After 14 and 21 days mice were treated with 100 µg/40 µL HDM i.t. 72 h after the last treatment 300 µm lung sections of agarose filled lungs were prepared. Staining with anti-RORγt (red), anti-CD3 (turquoise) and anti-TCRβ (green) allowed discrimination of CD3^+^TCRβ^+^ αβ T cells **(A)**, CD3^+^TCRβ^-^ non-conventional T cells **(B)** and CD3^-^TCRβ^-^ ILC3s **(C)**. Stained slices were evaluated with laser scanning confocal microscopy generating Z-Stacks. **(D)** A representative overview of a large airway is shown. Frequencies of αβ T cells, non-conventional T cells (nc T) and ILC3s among RORγt^+^ cells were determined in total lungs **(E)** around large **(F)** and small airways/blood vessels **(G)**. Abbreviations: AW, airway. Data are representative of at least four independent experiments. Scatter plot with mean ± SEM. n = 4-5 mice. Each dot represents the Z-stack of one mouse.

As we observed an increased frequency of IL-17A-producing pulmonary non-conventional T cells *in vivo* in HDM-exposed offspring of Abx-exposed mothers, we next determined whether IL-17A^+^ non-conventional T cells were increased in specific subregions within the lung. To this end, we combined the methods to determine pulmonary subregions based on autofluorescence (36), with *in situ* IHC staining of CD3 and TCRβ. Using this approach, we were able to discriminate αβ T cells (CD3^+^TCRβ^+^) (**Figure 5A**) and non-conventional T cells (CD3^+^TCRβ^-^) (**Figure 5B**) and ILCs (CD3^-^TCRβ^-^) (**Figure 5C**) expressing RORγt in lung sections and to assign them specifically to pulmonary subregions comprising large airways and small airways/blood vessels. Consistent with our flow based studies, the majority of RORγt^+^ cells in HDM-exposed animals were αβ T cells (61 %) (**Figures 5D** **, E**) whereas non-conventional T cells and ILCs represented only minor RORγt^+^ cell fractions with a frequency of 31 % and 7 %, respectively. This distribution was not affected by prenatally induced dysbiosis. Further, a similar distribution of RORγt^+^ cells was seen around larger airways (**Figure 5F**) as well as smaller airways/blood vessels (**Figure 5G**), and prenatal Abx treatment had no impact on the subpopulation assignment. Altogether, these experiments revealed no impact of prenatal Abx treatment on the distribution of subpopulations of pulmonary RORγt^+^ cells *in situ*.

### Prenatally induced dysbiosis of dams does not influence the frequency of contacts between RORγt^+^ cells and CD11c^+^ cells

**Figure 6:**
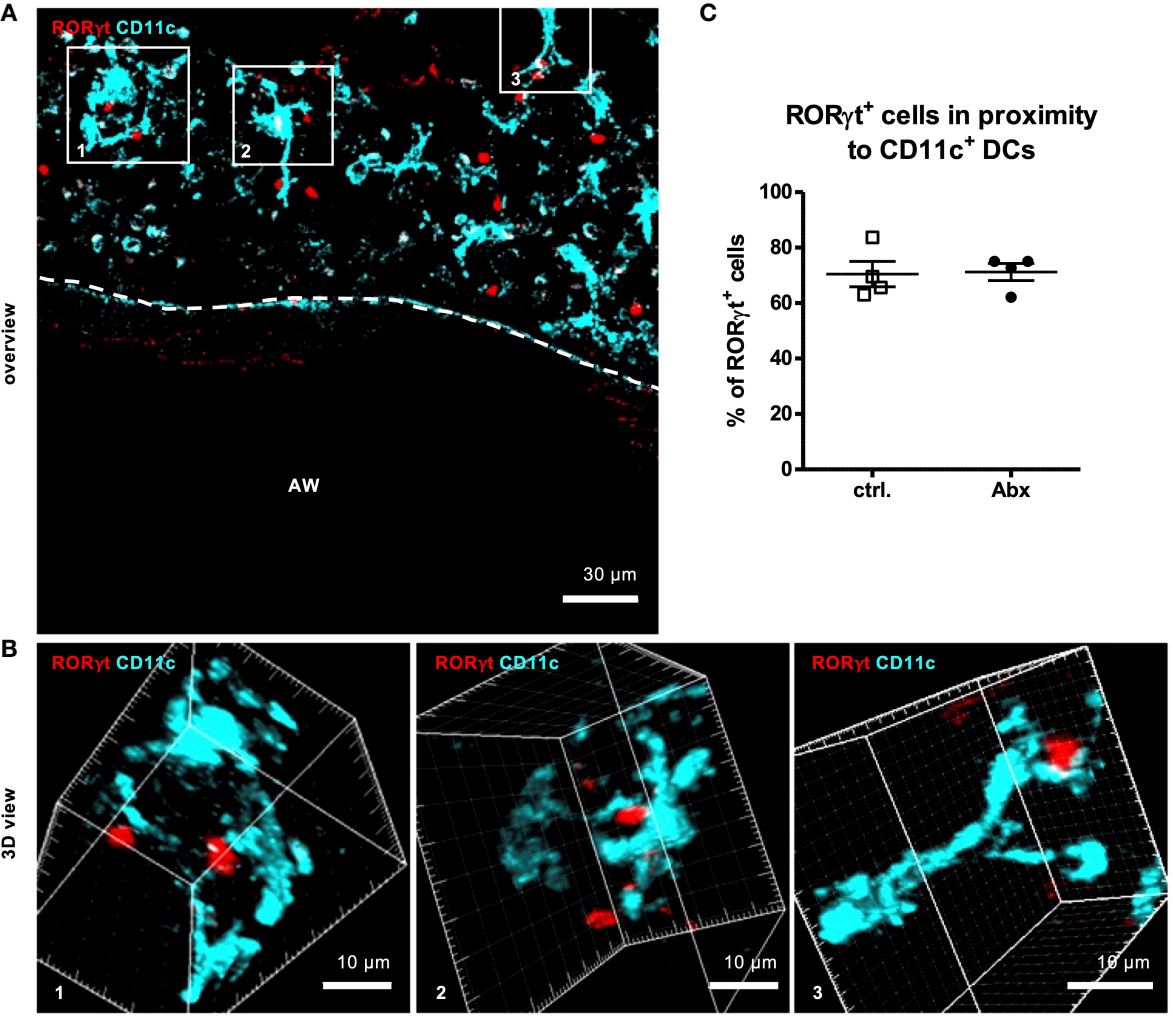
Prenatal antibiotic exposure of dams does not influence the frequency of contacts between RORγt^+^ cells and CD11c^+^ cells *in situ*. C57BL/6 mice were treated with vancomycin, ampicillin and gentamycin (all at 1.0 mg/mL)(Abx) from prenatal day 15 until birth. Mice treated with sucralose served as controls (ctrl.). At 6-8 weeks, offspring C57BL/6 mice were immunized i.p. with 10 µg/100 µL of HDM. Seven days later mice received an additional i.p. injection of HDM. After 14 and 21 days mice were treated with 100 µg/40 µL HDM i.t. 72 h after the last treatment 300 µm lung sections of agarose filled lungs were prepared. Staining with anti-RORγt (red) and anti-CD11c (turquoise) allowed evaluation of interactions between RORγt^+^ cells and CD11c^+^ DCs. Stained slices were evaluated with laser scanning confocal microscopy generating Z-Stacks. **(A)** A representative overview of a large airway is shown. Enlarged 3D views **(B)** of framed areas allowed 3D analysis of contact rates of RORγt^+^ cells with CD11c^+^ DCs **(C)**. Abbreviations: AW, airway. Data are representative of at least four independent experiments. Scatter plot with mean ± SEM. n = 4 mice. Each dot represents the Z-stack of one mouse.

As T cell activation requires prior interaction with proinflammatory dendritic cells (DC), we analyzed the *in situ* interaction between RORγt-expressing cells and CD11c^+^ DCs as was previously also shown for lung ILC3s (34). 3D-analyses of Z-stacks including analyses of each 1 µm thick slice of the Z-stack revealed that the majority of 71 % of RORγt^+^ cells were in close contact (<15 nm) with CD11c^+^ DCs (**Figures 6A** **–C**). Perinatal Abx treatment did not affect the frequency of RORγt^+^ cells interacting with CD11c^+^ cells (**Figure 6C**). Collectively these data suggest that while prenatal allergen exposure may influence initial seeding of the lung with non-conventional T cells after HDM exposure, it has little impact on asthma severity later in life. This study thus excludes the prenatal time window as a particularly critical window for maternal antibiotic exposure in mice.

## Discussion

Events that occur in fetal and early postnatal life influence the development of tolerance and long lasting risk of inflammatory diseases like asthma (34, 37, 38). However, the relative importance of pre- and postnatal time points critical for development of tolerance remain unclear. Herein, we address whether dysbiosis induced by prenatal Abx-treatment of dams evokes long lasting effects on asthma severity in offspring. Therefore, we exposed dams to a combination of ampicillin, gentamycin and vancomycin, which are commonly used to treat pregnant woman and newborn infants (39). A previous publication using this treatment already described the dysbiosis 4 days after birth by a 10-fold decrease of 16s rDNA copies/mg intestinal content and a change in microbial composition. Strongest effects were seen for Lactobacillales and Enterobacteriales being reduced in numbers upon Abx treatment and Actinomycetales and Bacteroida which were increased in numbers. Dysbiotic colonization showed a trend towards eubiosis already at the end of the window of investigation after 14 days (34). Using an equal treatment approach in the same animal facility, we demonstrate that prenatally induced dysbiosis was associated with increased numbers of non-conventional T cells with a potential for IL-17A-production following allergen sensitization and challenge. Knowing that γδ T cells, that belong together with invariant NKT cells and mucosal-associated invariant T cells to the IL-17A-producing non-conventional T cell compartment, and ILC3s develop between day 10 and 14 of pregnancy, whereas αβ T cells develop only after birth, the increase of non-conventional T cells within the IL-17A^+^ population appears consistent in a model of prenatally induced dysbiosis (40, 41). However, this modest observed increase in frequency of IL-17A^+^ non-conventional T cells does not alter asthma severity, as characterized by airway resistance, pro-asthmatic Th2/Th17 cytokine production, pulmonary localization and cell-cell contacts. Thus, our findings indicate that prenatal, maternal dysbiosis-related immune-modulation does not impact overall magnitude of asthma responses. However, given that in our model of experimental allergen-induced asthma, significant airway dysfunction is observed in all allergen-exposed animals (i.e. penetrance of asthma-like disease is 100 %), we cannot fully exclude the possibility that prenatal (maternal) dysbiosis might alter risk of asthma development in a less penetrant model of asthma-like disease.

The newborn lung harbors a Th2-biased immunological environment dominated by Th2 cells, ILC2s, eosinophils and basophils accumulating in the lung in an IL-33-dependent manner (42, 43). After birth, in mice within the first four weeks of life, this balance between Th cells shifts from the Th2 dominant first wave towards a Th1 phenotype and upregulation of regulatory T cells (Treg) (44, 45). In the context of allergic asthma, the Th1 shift appears relevant as asthma is a Th2-driven maladaptive immune response towards harmless aeroallergens. It is posited that intestinal commensals essentially drive the physiological Th2/Th1 conversion of the immune system (44). Thus, changes in the microbiome have the potential to modulate asthma severity by consolidating or alleviating the pro-asthmatic Th2 response or to alter asthma development/risk by altering the development of tolerogenic processes that naturally occur in the lung. Indeed, previous reports have demonstrated that Abx treatment worsens asthma severity in later life (37, 38). Mice treated with Abx peri- and postnatally (start of gestation till the end of the experiment) or solely postnatally (3-6 weeks) developed more severe asthma in OVA- or HDM-induced asthma models (37, 38). This observation correlated with reduced microbial diversity including loss of pro-regulatory organisms. The underlying mechanism relied on reduced Treg numbers in response to reduced *Clostridium* species (38, 45). Surprisingly then, administration of Abx did not affect asthma severity in our model. However, our study profoundly differs from these studies by solely investigating prenatal Abx treatment. Although we include in our considerations that the impact of antibiotic exposures persists into the early postnatal period (as reported previous (34)) when the lung and immune system continue to develop, our study focuses on earlier developmental windows than previous studies. To date, one additional study investigated prenatal Abx administration and the impact on asthma severity. Vancomycin was administered at different doses to dams. This treatment changed the gut microbiome in both mothers and pups and increased later life asthma severity in pups (46). Accordingly, we used vancomycin complemented with gentamycin and ampicillin to induce dysbiosis. Major experimental differences existed in the model allergen used for asthma induction. While Alhasan et al. (46) used ovalbumin without further adjuvant for asthma induction, our study used HDM as an allergen that better relates to human asthma. Comparing cellular influx into the lung, as indicated by BAL cells, HDM induces a much stronger allergic response than the OVA/adjuvant-free model. Likely, the strong asthma induction by HDM overwhelms the regulatory effects of Abx dosage. In combination with the naturally given differences between cohort microbiomes in different animal facilities, this may explain the different experimental outcomes.

Although we found increased numbers of IL-17A^+^ cells, particularly non-conventional T cells, in HDM-exposed offspring of Abx-treated dams, we were not able to detect any differences in cytokine production - either at baseline or following HDM restimulation - in lung cells isolated from offspring of sucralose or Abx-exposed dams by ELISA. A possible explanation relies on methodological differences. The frequency of IL-17A^+^ non-conventional T cells was assessed after *ex vivo* PMA/ionomycin stimulation while Th17-associated protein production was assessed after 72 hours of *in vitro* HDM restimulation. In detail, the former method aims at mostly stimulating allergen-specific αβ T cells (31, 32). As we do not actually see changes in the frequency of IL-17A^+^ conventional CD4^+^ T cells, this discrepancy may represent a technical limitation of *ex vivo* allergen restimulation as it is unclear to what extent pulmonary non-conventional T cells are activated by HDM in *ex vivo* cell cultures. In contrast, as flow-based methods for identification of IL-17^+^ producing cells utilize polyclonal stimulation (PMA/Ionomycin) it is also conceivable that the observed increase in IL-17A-producing non-conventional T cells represents expansion of non-HDM-specific non-conventional T cells that are exclusively captured using polyclonal stimulation techniques.

The mechanisms that might regulate altered non-conventional T cell recruitment to the lungs remain unclear, however, it is to be expected that dysbiosis induces changes in the maternal microbiome. Altered microbes or microbial metabolites might be retained by maternal IgG, and subsequently co-migrate to the uterus. After passage to the fetal intestine, they might lead to altered frequencies of non-conventional T cells. Such transplacental regulation was already demonstrated for ILC3s (47) where bacterial metabolites transferred from mother to offspring were able to regulate expression of antimicrobial peptides and influence microbial colonization in the fetal gut. While much of this work focused on changes in gut of offspring, recruitment of ILC3s to the neonatal lung were shown to be regulated by gut microbes. Further, these ILC3s had major implications for susceptibility to neonatal pneumonia (34). Specifically, CD103^+^CD11b^+^ DCs that capture antigen from gut commensal bacteria induced CCR4 expression on gut ILC3s licensing them for migration to the lung as well. This effect was diminished in Abx-treated mice leading to more severe pneumonia (34). Even γδ T cells that are part of the non-conventional T cells subpopulation were already shown to be regulated by antibiotic exposure, albeit the underlying experiment relied on postnatal antibiotic exposure and investigated long lasting effects on psoriasis (48). Altogether, these studies are consistent with our observation that early life shifts in microbiota induced by antibiotic exposure can cause dysregulated accumulation of IL-17 producing cells that may contribute to altered development of inflammatory diseases later in life.

Considering the reported changes in lung homing of ILC3s in response to prenatally induced dysbiosis and our observations that prenatally induced dysbiosis upregulated non-conventional T cells, we aimed at investigating effects on cellular distribution and interaction in the adult lung *in situ* (34, 49). This is of particular interest as T cell activation occurs *via* two pathways. It either involves MHC-mediated antigen-specific contacts with innate immune cells such as DCs accompanied by IL-6 secretion comparable to the pneumonia model and/or antigen-unspecific cell contact-independent cytokine secretion of IL-23 (50–52). Observing cellular contacts *in situ* allows for discrimination between these pathways. To assess cellular distribution we established an IHC staining approach enabling identification of potentially IL-17-producing cells in lung slices using the transcription factor RORγt (53). Staining with GATA3 to exclude Th2 cells as well as Hoechst staining to prove nuclear localization of the transcription factor RORγt validated this staining approach. *In situ* IHC analyses did not reproduce the observed increase of IL-17A^+^ cells in HDM-exposed offspring of antibiotic treated mothers as observed by FACS. Possibly, PMA/ionomycin stimulation, as used in the FACS approach, serves as a strong stimulant of IL-17A expression that may overcome a more homeostatic RORγt expression probed by IHC. Moreover, Yang et al. suggest that RORα^+^RORγt^-/-^ cells may have some residual IL-17A-producing capacity (54), suggesting that RORγt-expression may not serve as a perfect surrogate for IL-17A expression. We were unable to examine the distribution of RORα-expressing cells in our models. Nonetheless, we observed localization of RORγt^+^ cells in HDM-exposed offspring mostly close to large and only to a lower extent around small airways/blood vessels *via* IHC. In general, this localization is in accordance with the described localization of pulmonary immune cell populations (55). Further, it seems plausible that IL-17A^+^ cells accumulate around larger airways as they contribute to regulation of mucus production which was shown to occur mainly at this localization. Looking at the specific distribution of RORγt subpopulations (αβ T cells, non-conventional T cells and ILCs) within the HDM-exposed lung, revealed that dysbiosis did not affect cellular localization. Since the effects of IL-17A could also depend on activation instead of localization of RORγt^+^ cells, we determined the contact with CD11c^+^ DCs. RORγt^+^ cells were, independent of prior dysbiosis, mostly localized in close proximity with CD11c^+^ cells. Since activation of RORγt^+^ cells not only depends on cell-cell contact but also on IL-23 stimulation future studies will have to address this question in the context of prenatally induced dysbiosis.

In summary, our study revealed that solely prenatally induced dysbiosis exerts only minor effects on asthma severity later in life. To date, we cannot estimate whether this is a general observation or whether it rather is related to the individual microbiome of the experimental cohort or translationally the individual asthma patient. Thereby it emphasizes the need of individual considerations (e.g. dietary habit, microbial colonization, medication) in the context of microbiome regulated diseases. Further, it may help to direct future research on dysbiosis and its effects on allergic asthma to the postnatal time window. It also underlines the findings of existing studies that found major contribution of perinatal and postnatal factors such as mode of birth, lactation and postnatal contacts with microbiota in establishing a healthy commensal repertoire and subsequently contributing to appropriate barrier formation and alveolarization in the lung. By showing that prenatally induced dysbiosis affects IL-17^+^ cells in the offspring, we qualify IL-17-mediated inflammatory processes as the most sensitive and at first regulated processes in response to early dysbiosis.

## Data availability statement

The raw data supporting the conclusions of this article will be made available by the authors, without undue reservation.

## Ethics statement

The animal study was reviewed and approved by Schleswig-Holstein state authorities and CCHMC Institutional Animal Care and Use Committee.

## Author contributions

All authors revised the manuscript. IL performed the majority of the experiment in Cincinnati and Lübeck, and analyzed the data. AW, JH, and JM assisted with experiments in Cincinnati. YL critically discussed the data. CC and HD contributed to study design and data discussion. PK designed the research, supervised and took the responsibility of all experiments performed in Lübeck. IPL designed the research, supervised and took the responsibility of all experiments performed in Cincinnati. IS discussed the data and wrote the manuscript. All authors contributed to the article and approved the submitted version.

## Funding

This project was supported by Deutsche Forschungsgemeinschaft Grants IRTG 1911 (project A2 to PK), NHLBI R01 HL122300 (to IPL), and NHLBI R01 HL149366 (to IPL).

## Conflict of interest

The authors declare that the research was conducted in the absence of any commercial or financial relationships that could be construed as a potential conflict of interest.

## Publisher’s note

All claims expressed in this article are solely those of the authors and do not necessarily represent those of their affiliated organizations, or those of the publisher, the editors and the reviewers. Any product that may be evaluated in this article, or claim that may be made by its manufacturer, is not guaranteed or endorsed by the publisher.
