# Supplementary material for: Prenatal antibiotics exposure does not influence experimental allergic asthma in mice
Source: Front Immunol. 2022 Aug 10;13:937577. doi: 10.3389/fimmu.2022.937577 (PMC9399857; doi:10.3389/fimmu.2022.937577)
Supplement: Supplementary file 1 [file Presentation_1.pptx]

## Slide 1
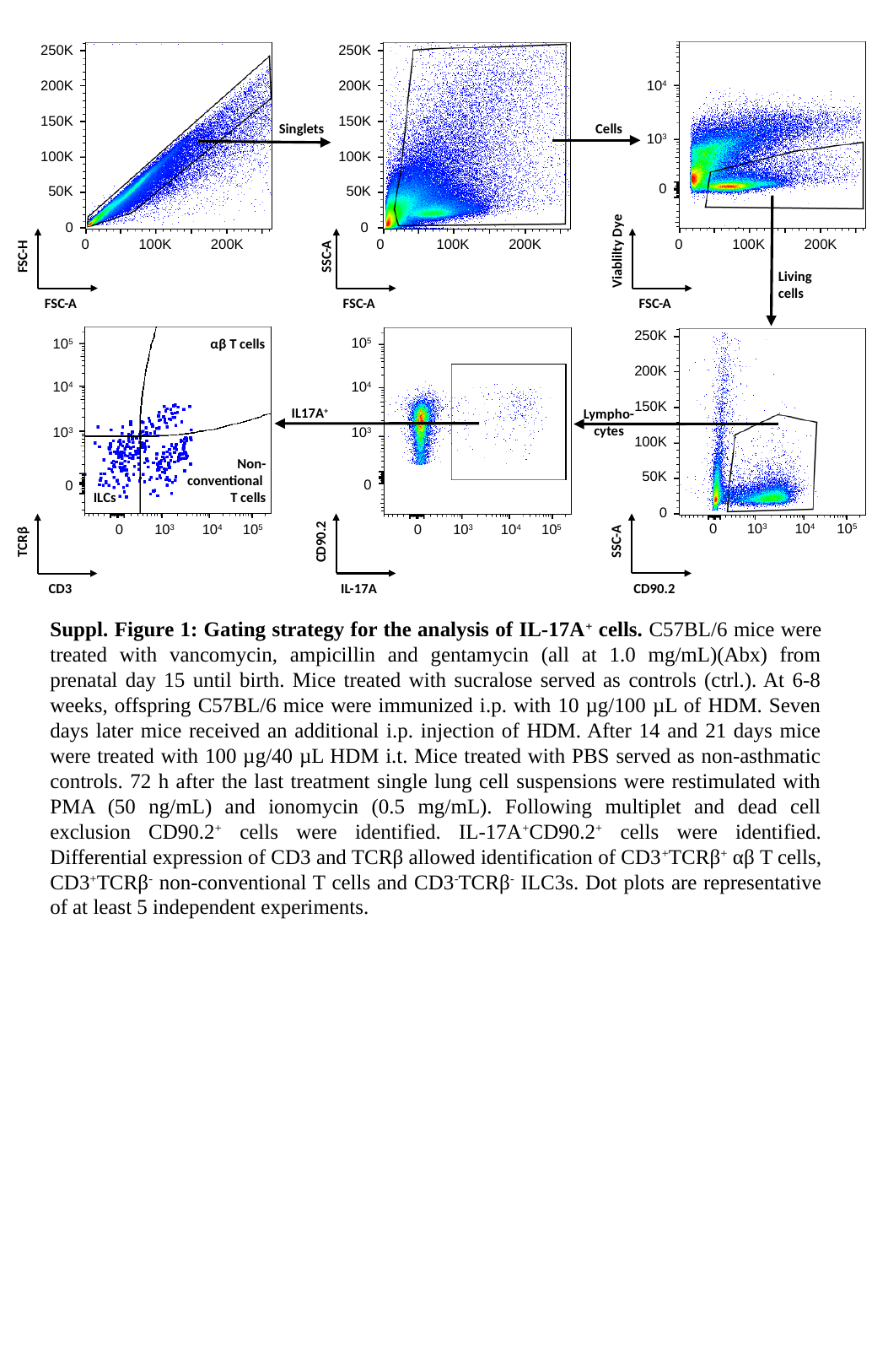

250K
250K
200K
200K
104
150K
150K
Singlets
Cells
103
100K
100K
0
50K
50K
0
0
0
100K
200K
0
100K
200K
0
100K
200K
Viablilty Dye
FSC-H
SSC-A
Living
cells
FSC-A
FSC-A
FSC-A
250K
105
105
αβ T cells
200K
104
104
150K
IL17A+
Lympho-cytes
103
103
100K
Non-conventional
T cells
50K
0
0
ILCs
0
0
103
104
105
0
103
104
105
0
103
104
105
SSC-A
CD90.2
TCRβ
CD90.2
IL-17A
CD3
Suppl. Figure 1: Gating strategy for the analysis of IL-17A+ cells. C57BL/6 mice were treated with vancomycin, ampicillin and gentamycin (all at 1.0 mg/mL)(Abx) from prenatal day 15 until birth. Mice treated with sucralose served as controls (ctrl.). At 6-8 weeks, offspring C57BL/6 mice were immunized i.p. with 10 µg/100 µL of HDM. Seven days later mice received an additional i.p. injection of HDM. After 14 and 21 days mice were treated with 100 µg/40 µL HDM i.t. Mice treated with PBS served as non-asthmatic controls. 72 h after the last treatment single lung cell suspensions were restimulated with PMA (50 ng/mL) and ionomycin (0.5 mg/mL). Following multiplet and dead cell exclusion CD90.2+ cells were identified. IL-17A+CD90.2+ cells were identified. Differential expression of CD3 and TCRβ allowed identification of CD3+TCRβ+ αβ T cells, CD3+TCRβ- non-conventional T cells and CD3-TCRβ- ILC3s. Dot plots are representative of at least 5 independent experiments.

## Slide 2
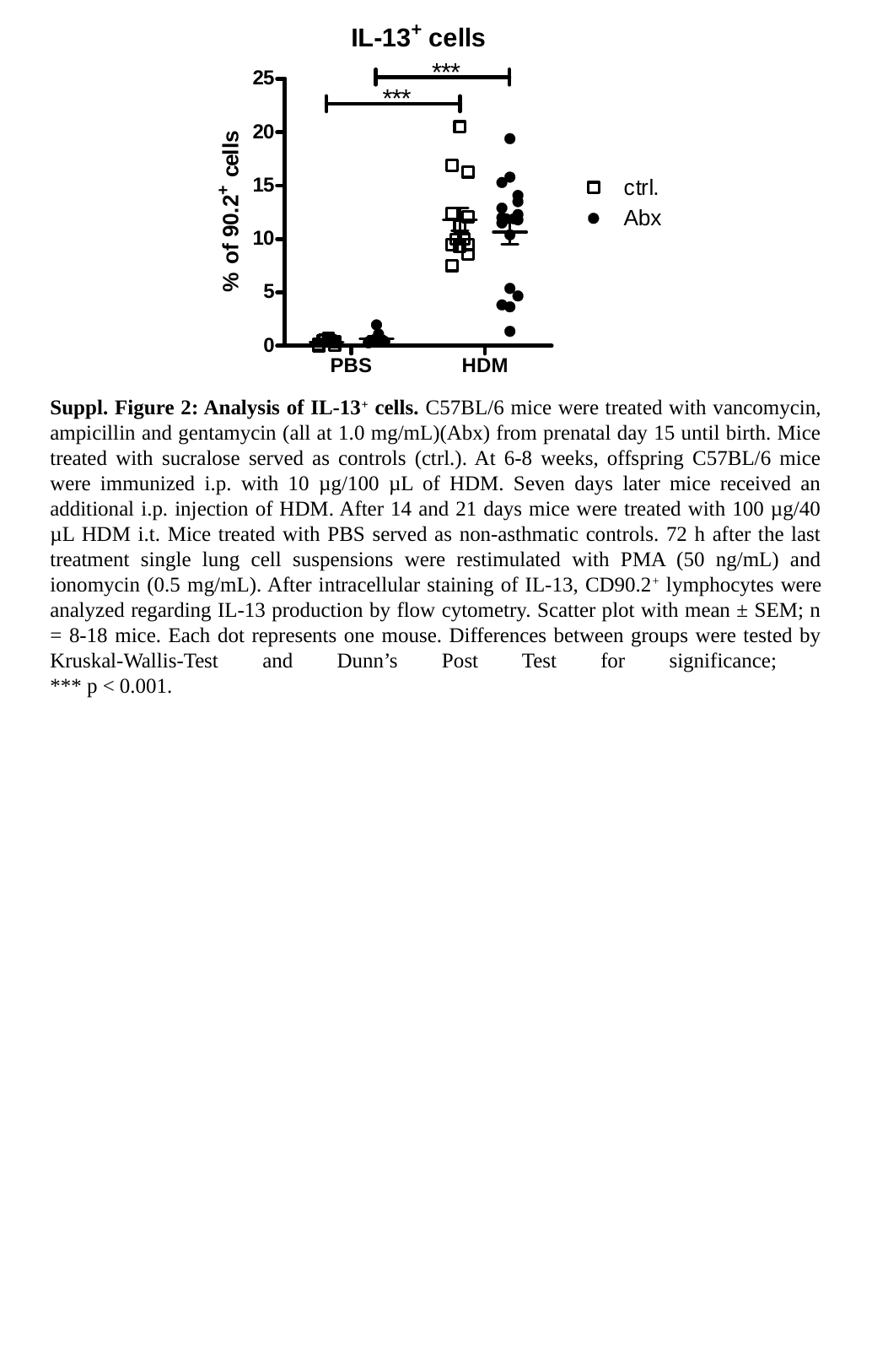

Suppl. Figure 2: Analysis of IL-13+ cells. C57BL/6 mice were treated with vancomycin, ampicillin and gentamycin (all at 1.0 mg/mL)(Abx) from prenatal day 15 until birth. Mice treated with sucralose served as controls (ctrl.). At 6-8 weeks, offspring C57BL/6 mice were immunized i.p. with 10 µg/100 µL of HDM. Seven days later mice received an additional i.p. injection of HDM. After 14 and 21 days mice were treated with 100 µg/40 µL HDM i.t. Mice treated with PBS served as non-asthmatic controls. 72 h after the last treatment single lung cell suspensions were restimulated with PMA (50 ng/mL) and ionomycin (0.5 mg/mL). After intracellular staining of IL-13, CD90.2+ lymphocytes were analyzed regarding IL-13 production by flow cytometry. Scatter plot with mean ± SEM; n = 8-18 mice. Each dot represents one mouse. Differences between groups were tested by Kruskal-Wallis-Test and Dunn’s Post Test for significance;
*** p < 0.001.

## Slide 3
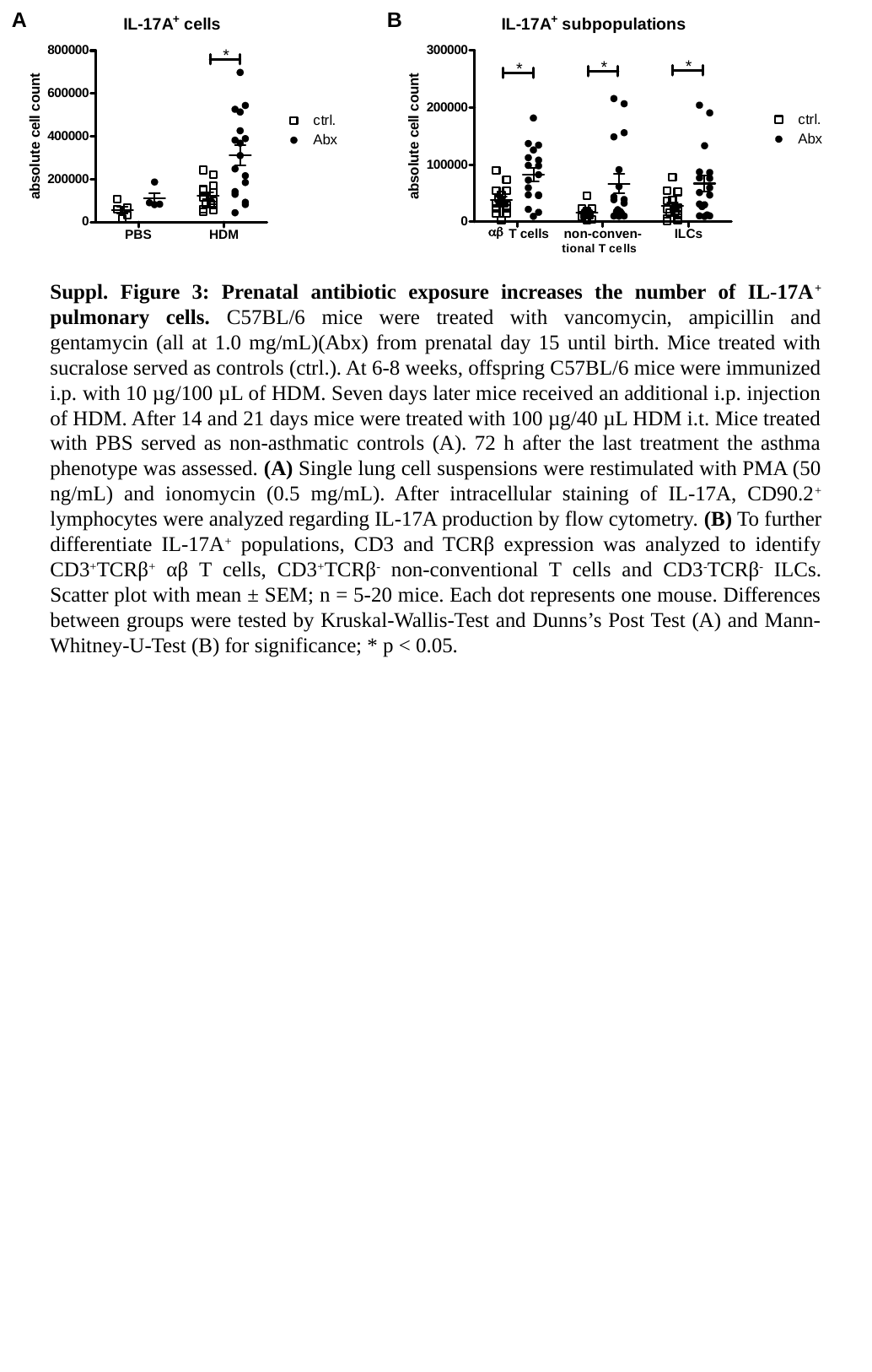

A
B
Suppl. Figure 3: Prenatal antibiotic exposure increases the number of IL-17A+ pulmonary cells. C57BL/6 mice were treated with vancomycin, ampicillin and gentamycin (all at 1.0 mg/mL)(Abx) from prenatal day 15 until birth. Mice treated with sucralose served as controls (ctrl.). At 6-8 weeks, offspring C57BL/6 mice were immunized i.p. with 10 µg/100 µL of HDM. Seven days later mice received an additional i.p. injection of HDM. After 14 and 21 days mice were treated with 100 µg/40 µL HDM i.t. Mice treated with PBS served as non-asthmatic controls (A). 72 h after the last treatment the asthma phenotype was assessed. (A) Single lung cell suspensions were restimulated with PMA (50 ng/mL) and ionomycin (0.5 mg/mL). After intracellular staining of IL-17A, CD90.2+ lymphocytes were analyzed regarding IL-17A production by flow cytometry. (B) To further differentiate IL-17A+ populations, CD3 and TCRβ expression was analyzed to identify CD3+TCRβ+ αβ T cells, CD3+TCRβ- non-conventional T cells and CD3-TCRβ- ILCs. Scatter plot with mean ± SEM; n = 5-20 mice. Each dot represents one mouse. Differences between groups were tested by Kruskal-Wallis-Test and Dunns’s Post Test (A) and Mann-Whitney-U-Test (B) for significance; * p < 0.05.

## Slide 4
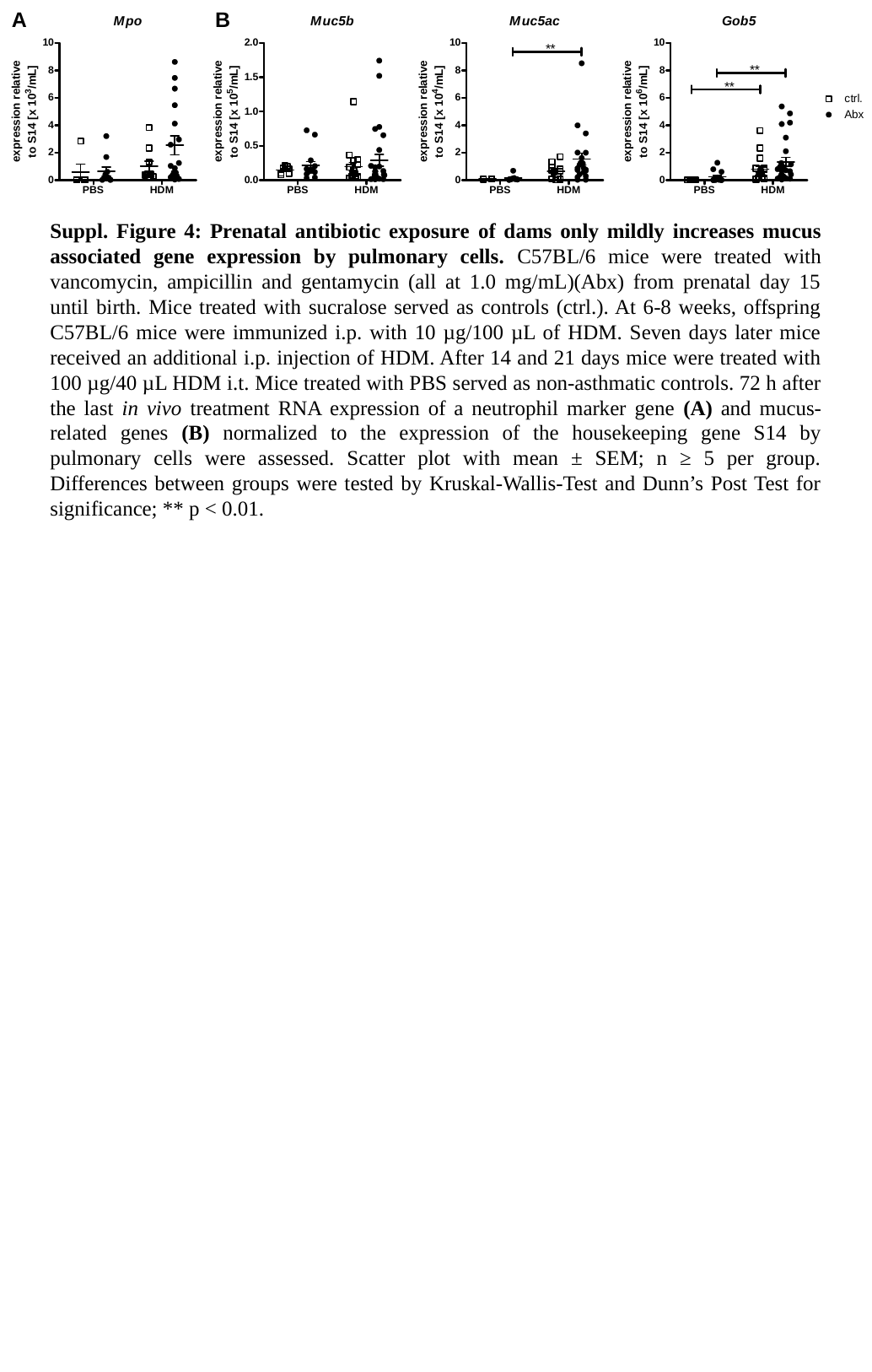

A
B
Suppl. Figure 4: Prenatal antibiotic exposure of dams only mildly increases mucus associated gene expression by pulmonary cells. C57BL/6 mice were treated with vancomycin, ampicillin and gentamycin (all at 1.0 mg/mL)(Abx) from prenatal day 15 until birth. Mice treated with sucralose served as controls (ctrl.). At 6-8 weeks, offspring C57BL/6 mice were immunized i.p. with 10 µg/100 µL of HDM. Seven days later mice received an additional i.p. injection of HDM. After 14 and 21 days mice were treated with 100 µg/40 µL HDM i.t. Mice treated with PBS served as non-asthmatic controls. 72 h after the last in vivo treatment RNA expression of a neutrophil marker gene (A) and mucus-related genes (B) normalized to the expression of the housekeeping gene S14 by pulmonary cells were assessed. Scatter plot with mean ± SEM; n ≥ 5 per group. Differences between groups were tested by Kruskal-Wallis-Test and Dunn’s Post Test for significance; ** p < 0.01.

## Slide 5
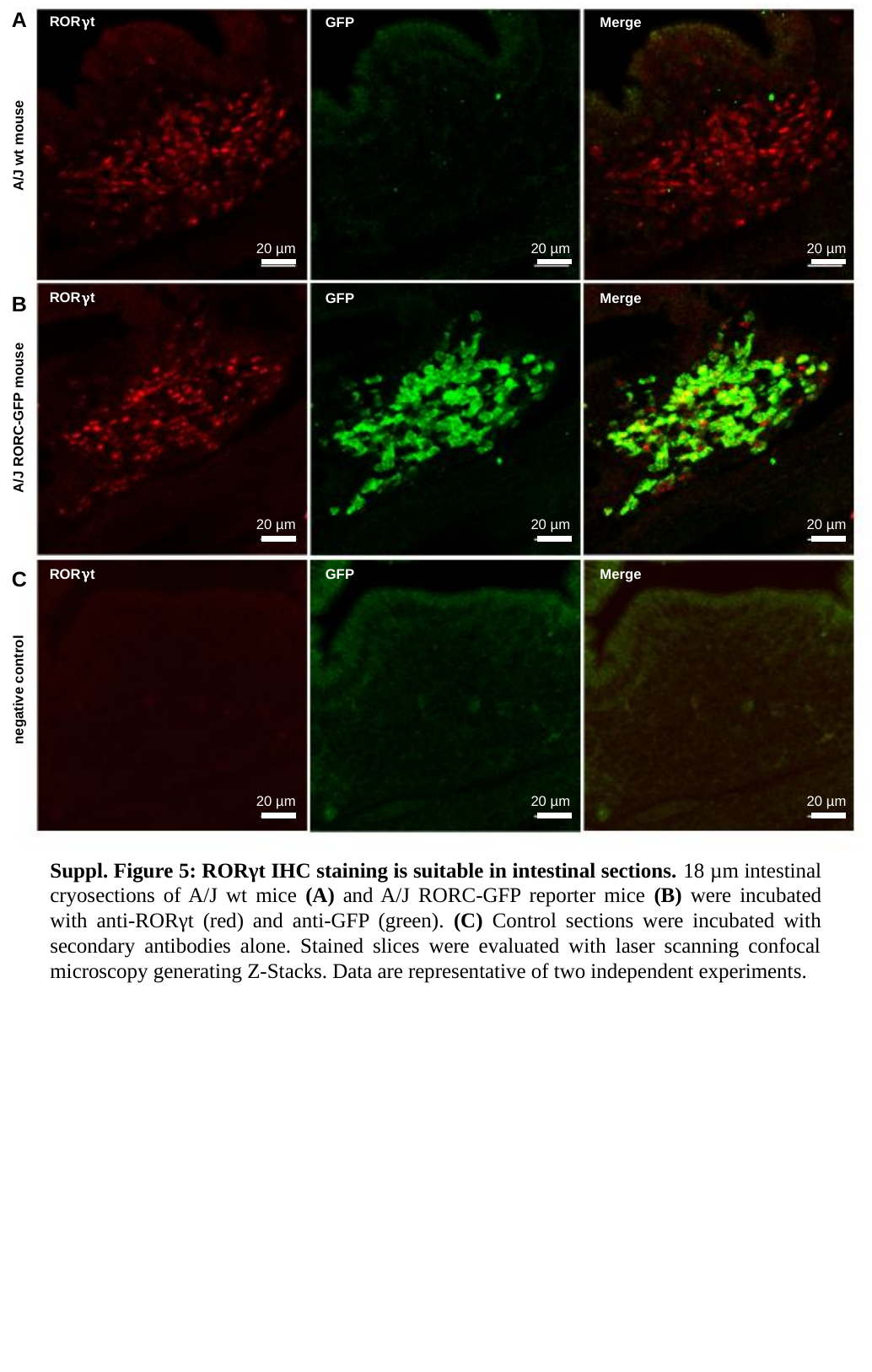

A
RORγt
GFP
Merge
A/J wt mouse
20 µm
20 µm
20 µm
RORγt
GFP
Merge
B
A/J RORC-GFP mouse
20 µm
20 µm
20 µm
RORγt
GFP
Merge
C
negative control
20 µm
20 µm
20 µm
Suppl. Figure 5: RORγt IHC staining is suitable in intestinal sections. 18 µm intestinal cryosections of A/J wt mice (A) and A/J RORC-GFP reporter mice (B) were incubated with anti-RORγt (red) and anti-GFP (green). (C) Control sections were incubated with secondary antibodies alone. Stained slices were evaluated with laser scanning confocal microscopy generating Z-Stacks. Data are representative of two independent experiments.

## Slide 6
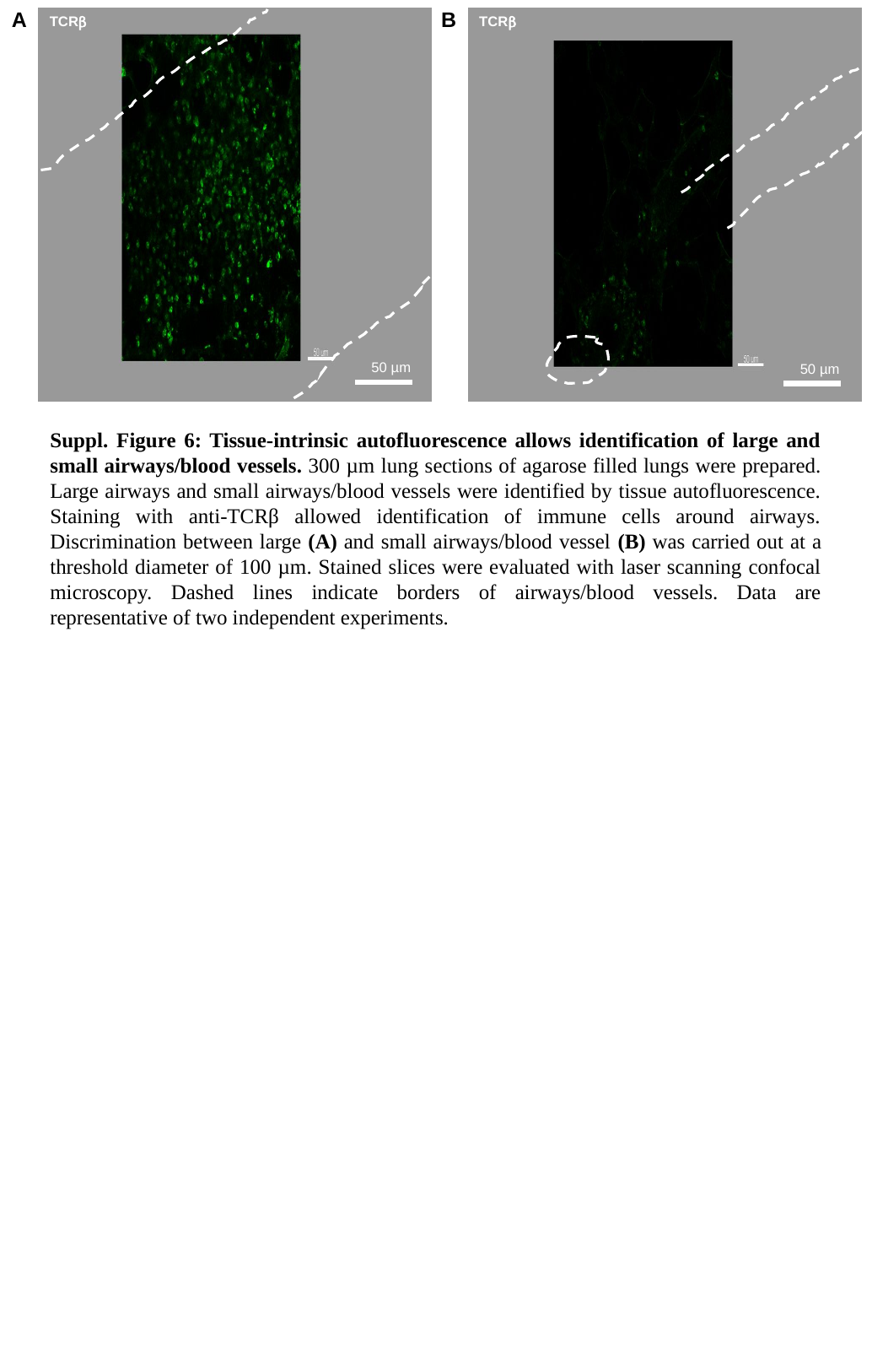

A
B
TCRβ
TCRβ
50 µm
50 µm
Suppl. Figure 6: Tissue-intrinsic autofluorescence allows identification of large and small airways/blood vessels. 300 µm lung sections of agarose filled lungs were prepared. Large airways and small airways/blood vessels were identified by tissue autofluorescence. Staining with anti-TCRβ allowed identification of immune cells around airways. Discrimination between large (A) and small airways/blood vessel (B) was carried out at a threshold diameter of 100 µm. Stained slices were evaluated with laser scanning confocal microscopy. Dashed lines indicate borders of airways/blood vessels. Data are representative of two independent experiments.
